# Higher prevalence of kidney function impairment among older people living with HIV in Uganda

**DOI:** 10.21203/rs.3.rs-4364155/v1

**Published:** 2024-05-13

**Authors:** Amutuhaire Judith Ssemasaazi, Robert Kalyesubula, Yukari C Manabe, Phoebe Mbabazi, Susan Naikooba, Faizo Ssekindi, Esther Nasuuna, Pauline Byakika Kibwika, Barbara Castelnuovo

**Affiliations:** Makerere University College of Health Sciences; Makerere University College of Health Sciences; Infectious Diseases Institute; Infectious Diseases Institute; Infectious Diseases Institute; Infectious Diseases Institute; Infectious Diseases Institute; Makerere University College of Health Sciences; Infectious Diseases Institute

**Keywords:** Older persons, kidney function impairment, sub-Saharan Africa

## Abstract

**Background:**

People living with HIV (PLWH) are at risk of kidney function impairment due to HIV-related inflammation, antiretroviral therapy (ART), diabetes mellitus, and hypertension. Older persons may experience a higher burden of chronic kidney disease (CKD) as kidney function declines with increasing age. There is a paucity of data comparing the prevalence of kidney function impairment in older PLWH to that in HIV-uninfected people in sub-Saharan Africa.

**Methods:**

We conducted a cross-sectional study among people aged ≥ 60 years living with and without HIV in Kampala, Uganda who were matched 1:1 by community location. We collected data on sociodemographics, comorbidities, and HIV-related clinical characteristics. We defined kidney function impairment as an estimated glomerular filtration rate(eGFR) < 60mls/min/1.73m^2^ with or without proteinuria. We constructed multivariable logistic regression models to study associations between participant characteristics and kidney function impairment.

**Results:**

We enrolled 278 people (median age 66 years); 50% were PLWH, and 51.8% were female. Overall, the prevalence of kidney function impairment was 23.0% (95% CI:18.4%−28.4%); 33.1% (95% CI: 25.7%−41.4%) versus 12.9% (95% CI: 8.3%−19.7%) among people living with and without HIV (p-value < 0.01). The prevalence of proteinuria among PLWH versus people without HIV was 43.9% (95% CI:35.8%−52.3%) versus 19.4% (95% CI:13.6%−26.9%) p-value < 0.01. Living with HIV (OR = 3.89(95% CI: 2.04–7.41), p-value < 0.01), older age (OR = 1.13, (95% CI:1.07–1.20), p-value < 0.01), female sex (OR = 1.95, (95% CI:1.06–3.62), p-value = 0.03) and a prior diagnosis of hypertension (OR = 2.19(95% CI:1.02–4.67), p-value = 0.04) were significantly associated with kidney function impairment.

**Conclusions:**

HIV infection is strongly associated with kidney function impairment among older PLWH. Prioritizing routine measurements of kidney function and proteinuria in older PLWH will enable early detection and institution of measures to reduce the progression of kidney disease.

## Background

Globally, access to antiretroviral therapy (ART) has averted at least 20 million HIV-related deaths over the last two decades; therefore, more people are aging with HIV [1]. Aging with HIV is associated with an increased risk of multimorbidity [2] such as noncommunicable diseases (NCDs) including chronic kidney disease (CKD) [3, 4]. Nephron senescence is a recognized age-related change that eventually leads to a decline in kidney function and increases the risk of CKD in older persons [5]. In people living with HIV (PLWH); aging, chronic inflammation which persists during suppressive ART, and nephrotoxic ART such as tenofovir disoproxil fumarate (TDF) [5–7] converge to increase the risk of CKD by threefold [8]. Moreover, traditional risk factors for CKD such as diabetes mellitus and hypertension are more prevalent among PLWH [2, 9]. Chronic kidney disease is a progressive disease that manifests with several indicators of kidney function impairment such as proteinuria [10]. Kidney failure is associated with increased morbidity, poor quality of life, and increased risk of death among PLWH [11–14]. In low-income countries (LICs) such as Uganda, there is limited access to life-saving dialysis and kidney transplants to manage end-stage kidney disease(ESKD)/kidney failure and even when available, the costs exceed the average income for most patients [15, 16]. Studies in the general population have documented the association of HIV with kidney disease in both low- and high-resource settings[17–20], but there is a paucity of data documenting the excess prevalence of kidney function impairment in older PLWH in Uganda and sub-Saharan Africa. We sought to determine the additional prevalence of kidney function impairment in older people living with HIV compared to those without HIV in Uganda.

## Methods

### Study design and setting

We conducted a cross-sectional study at the Infectious Diseases Institute (IDI) clinic in Mulago, Kampala, Uganda between April and August 2023. The IDI is an implementing partner for the President’s Emergency Plan for AIDS Relief (PEPFAR) with clinics in 18 districts in the country. The IDI flagship adult clinic at Mulago Hospital takes care of more than 8,000 HIV-infected patients and among these patients, more than 1000 are aged ≥ 60 years.

We consecutively enrolled PLWH aged ≥ 60 years who were attending the HIV clinic at IDI and people without HIV recruited within a 2-kilometer distance of the same communities where the participating PLWH lived. The participants were recruited on a 1:1 ratio. We defined older people as those aged ≥ 60 years per the United Nations definition [21, 22]. Participants were excluded if they could not provide blood or urine samples for study measurements.

### Data collection tools and procedures

We collected data using an electronic structured questionnaire developed in Research Electronic Data Capture (REDCap) developed by Vanderbilt University [23]. HIV-uninfected participants were counseled and tested for HIV before enrollment in the study.

For each participant, we collected sociodemographic data (sex, age in years, education level, income status), and anthropological measurements (height in meters and weight in kg) using calibrated scales and calculated the body mass index in kg/m^2^. We collected information on comorbidities (diabetes mellitus, hypertension, other cardiovascular disease, liver disease, and others), prescriptions and over-the-counter drugs, past or present history of smoking, history of current alcohol use, and history of HIV, which included duration of ART, antiretroviral drugs (previous and current regimens), history and duration of TDF use, most recent viral load and CD4 count. We measured blood pressure using a standardized automated Omron M2 basic blood pressure monitor with consistent positioning (patients were seated upright with back and arm support). We took three readings and calculated the average. We measured fasting blood sugar in mmol/l using a point-of-care calibrated on-call plus glucometer manufactured by ACON laboratories, USA. For participants with no prior history of diabetes mellitus, a diagnosis of diabetes mellitus was made if one had elevated blood glucose (nonfasting ≥ 11.1 mmol/L or fasting ≥ 7 mmol/L) in the presence of symptoms. If elevated values were found in someone asymptomatic; a repeat fasting blood sugar was performed on a subsequent day to confirm the diagnosis.

We collected an early morning urine sample from each patient for measurement of urine protein by dipstick urinalysis and a venous blood sample for measurement of serum creatinine. We defined kidney function impairment as an estimated glomerular filtration rate (eGFR) < 60 mls/min per 1.73 m^2^ with or without proteinuria on a dipstick urine test. This creatinine-based eGFR was calculated from the 2009 version of the chronic kidney disease epidemiology collaboration) (CKD-EPI) (CKD-EPI 2009) [24] without correcting for race [25, 26]. We used the CKD-EPI 2009 which is widely used in clinical practice and a recent study showed it to perform better than the race-free creatinine-based CKD-EPI 2021 equation in sub-Saharan Africa [27]. We categorized the stages of kidney function impairment by eGFR as stage 1:≥ 90, stage 2:≥60-<90, stage 3: ≥30-<60, stage 4:≥15-<30 and stage 5: <15mls/min/1.73m^2^ [28].

We measured proteinuria using Siemens Multistix GP Urine Test Strips and we categorized it as negative if no protein was detected or positive if at least trace protein (≥ 30mg/dl) was detected.

### Data Analysis

We exported the data to STATA version 14 (Stata Corp LLC, College Station, TX).

We reported the proportions of participants with kidney function impairment and proteinuria in both groups with 95% confidence interval (95% CI) and compared them with a chi-square test.

The additional prevalence of kidney function impairment due to HIV was reported as a difference between the proportions in the group of PLWH and people without HIV with its 95% CI and a p-value corresponding to the z-statistic for testing the significance of a difference in proportions between two groups.

We summarized other categorical variables as proportions and continuous variables as medians with interquartile ranges (IQR). Comparisons were based on a chi-square test for categorical variables otherwise Fisher’s exact test was used if any of the cells had ≤ 5 observations. Comparisons for continuous variables were based on the Wilcoxon rank sum test.

We performed logistic regression with HIV as the main exposure and performed a stratified analysis by HIV status to study associations between various participant characteristics and kidney function impairment. We considered the level of significance at a p-value ≤ 0.2 for bivariate analysis and p ≤ 0.05 for multivariate analysis. We assessed confounding factors by comparing unadjusted and adjusted models, interactions using the loglikelihood ratio test, and goodness of fit of the model using the Hosmer–Lemeshow test.

## Results

We enrolled 278 men and women aged ≥ 60 years; 50% were PLWH and 51.8% were female.

PLWH and those living without HIV were similar except for smoking status, use of non-HIV related medications, prior history of tuberculosis, median systolic and diastolic blood pressure, body mass index, and proteinuria (Table 1).

Among the 139 PLWH, the median CD4 count was 634 (IQR: 486–917) cells/mm^3^, 5.8% had a detectable viral load (> 50 copies/ml), 80.6% had WHO clinical stage 3 or 4 disease at the start of ART, 95.7% had ever used TDF in their ART regimen, and 66.2% were currently on a TDF-based regimen (Table 2).

### Burden of kidney function impairment among study participants

Overall, 23.0% (95% CI:18.4%−28.4%) had kidney function impairment. Among the PLWH, 33.1% (95% CI: 25.7%−41.4%) had kidney function impairment compared to 12.9% (95% CI: 8.3%−19.7%) among people without HIV, (p-value < 0.01); the additional prevalence of kidney function impairment in PLWH was 20.2% (95%CI: 10.6%−29.0%), p-value < 0.01.

**Figure 1:**
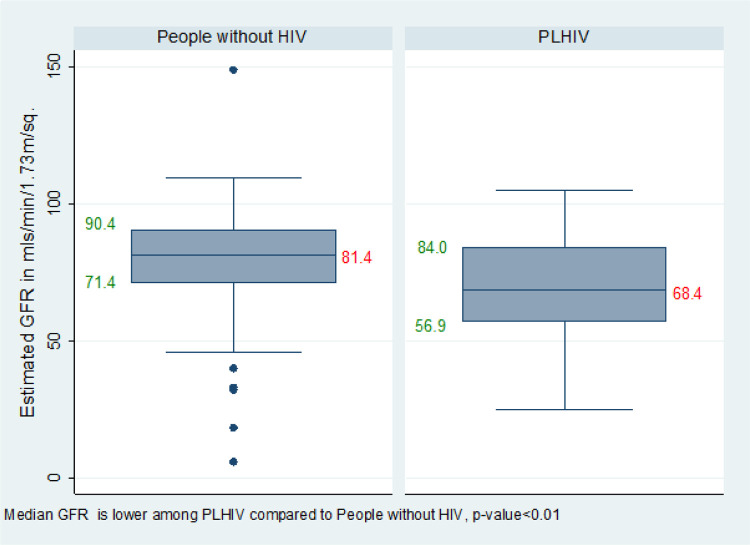
Median estimated GFR of people aged ≥60 years living with and without HIV in Uganda.

The median GFR among PLWH versus people without HIV was 68.4 (IQR:56.9–84.0) mls/min/1.73m2 versus 81.4 (IQR:71.4–90.4) mls/min/1.73m2, p-value < 0.01 (Fig. 1).

**Figure 2:**
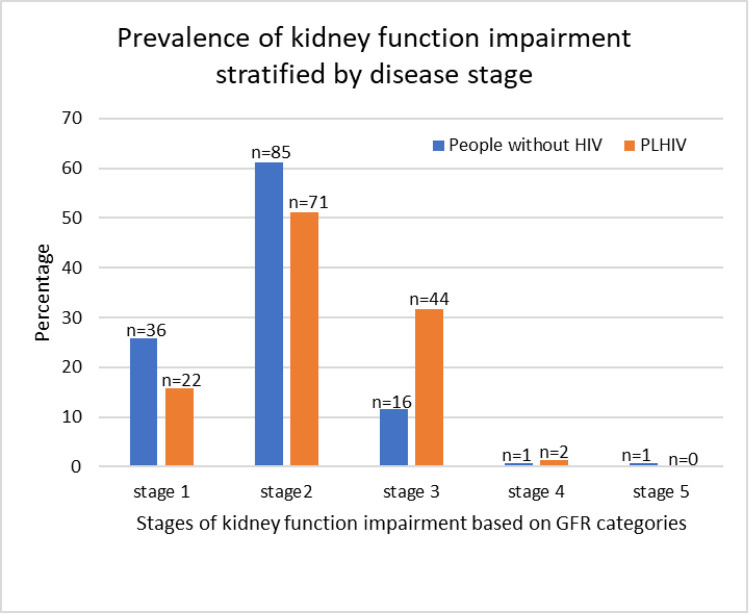
Prevalence of kidney function impairment among PLWH and people without HIV stratified by disease stage (GFR categories).

The prevalence of kidney function impairment among PLWH and people without HIV varied by disease stage. PLWH constituted the majority in stage 3 and stage 4 of kidney function impairment (Fig. 2).

Older PLWH had a higher prevalence of proteinuria 43.9% (95% CI:35.8–52.3) versus 19.4% (95% CI: 13.6–26.9) among older people without HIV, p-value < 0.01.

Older age (OR = 1.13, (95% CI: 1.07–1.20), p-value < 0.01), being female (OR = 1.95, (95% CI: 1.06–3.62), p-value = 0.03) and living with HIV (OR = 3.89, (95% CI: 2.04–7.41), p < 0.01) were associated with kidney function impairment at multivariate analysis (Table 3). Additionally, in a stratified analysis by HIV status, a prior diagnosis of hypertension (OR = 2.19, (95% CI:1.02–4.67), p-value = 0.04) was associated with kidney function impairment among older PLWH

## Discussion

Our study shows that one-third of the aging Ugandan HIV population had kidney function impairment that was significantly higher than the prevalence in community-matched HIV-uninfected controls. Data documenting the burden of kidney function impairment in aging HIV populations come mostly from high-income countries [8, 19, 29]. Overall, we found a high prevalence of kidney function impairment among people aged ≥ 60 years living with and without HIV in Uganda. Our data agree with studies that have shown that PLWH experience a high burden of kidney disease [19, 29, 30]. HIV infection significantly increases the odds of kidney function impairment [8, 9, 31, 32] and various forms of HIV-associated nephropathy have been documented in previous research[9, 33]. However, our study demonstrated an excess burden of kidney function impairment among older PLWH compared to previous prevalences reported in the general population of PLWH in Uganda[34, 35] and other LICs[18, 30, 36, 37]. These findings imply that there is a need for research and clinical care programs to evolve and prioritize kidney disease detection in the population of older PLWH.

The prevalence of proteinuria in our study was at least two times higher in PLWH than in people without HIV. Other studies such as the AGE_h_IV cohort study [38] have shown that the burden of albuminuria is higher among people living with HIV than in people without HIV. However, our study reported higher estimates of proteinuria than what has been reported in non-African settings [38, 39]. Previous studies have pointed to genetic risks for kidney injury such as the presence of the APOL1 gene which is unique to African populations and may account for racial disparities in the burden of CKD [40–43]. Proteinuria is a biomarker for kidney injury[28]; thus further research into context-specific risk factors for kidney disease is needed to understand the cause of kidney injury in older PLWH.

Kidney disease is progressive; thus these findings of the excess prevalence of kidney function impairment and proteinuria may have implications for the health and survival of old PLWH in a country where there is limited access to life-saving therapies such as dialysis and renal transplants for kidney failure [15, 16].

Increasing age and female sex were significantly associated with kidney function impairment in both PLWH and people without HIV. These findings are consistent with previous studies from Uganda and Africa where increasing age and female sex were associated with kidney disease in both PLWH and people without HIV [18, 31, 44, 45].

Among older PLWH, a prior diagnosis of hypertension significantly increased the odds of kidney function impairment. Hypertension is a known traditional risk factor for CKD [46] and hypertensive nephrosclerosis is a well-recognized pathological process that eventually leads to kidney failure [47]. A high prevalence of hypertension has been reported among aging PLWH [2, 48] and the association of such traditional risk factors with CKD is documented in previous studies [46, 49]. The implementation of blood pressure control programs in older PLWH and hypertension can benefit kidney health as the same approach has been shown to improve outcomes in other populations at risk of hypertension-related kidney injury such as people with cardiovascular diseases and diabetes mellitus[50].

In our study, HIV was the strongest significantly associated factor with kidney function impairment. Unlike other studies, we did not find an association between diabetes mellitus[29, 51] and kidney function impairment, smoking [9, 52, 53], body mass index (BMI), socioeconomic status (monthly household income) [44, 54] or prior diagnosis of tuberculosis [55] [56] as other studies did. Unlike these previous studies, in our study, fewer participants reported a history of smoking, and there were fewer diabetic patients. The monthly household income was not different between PLWH and people without HIV or between people with and without kidney function impairment. Likewise, the BMI was not different between people with and without kidney function impairment and all the people who had a previous diagnosis of tuberculosis were among PLWH.

Among the PLWH in our study, only a small proportion, 2.2%, had CD4 less than 200 cells per ml and only 5.8% had detectable viral loads (> 50copies/ml). Almost all (95.7%) of our PLWH had ever used TDF. These proportions may explain the lack of association between these variables and kidney function impairment in our study although previous studies have shown a significant association[8, 18, 57, 58]. In our study, we observed higher prevalence of kidney function impairment in participants on abacavir-based regimens; most of the participants on abacavir-based regimens had previously been on TDF, but were switched over due to low eGFR.

Previous studies on kidney function impairment among old people with HIV in Uganda and sub-Saharan Africa have not included uninfected controls. A strength of our study was a comparator group of older people without HIV from similar communities which allowed us to compare the characteristics between the two populations as well as the outcome and ascertain the excess prevalence of kidney function impairment attributable to HIV. A limitation of our study was that it was cross-sectional so we were unable to observe changes in kidney function over three months. In addition, we did not perform ultrasound scanning so we could not differentiate between acute and chronic kidney function impairment. We also used dipsticks to detect proteinuria which is a less specific method than the urine albumin to creatinine ratio (UACR).

## Conclusions

There is an excess prevalence of kidney function impairment amongolder PLWH with one-third of our aging PLWH having impaired kidney function. In LICs, early ascertainment of kidney function in older PLWH should be prioritized with routine measurement of urine protein and kidney function. Furthermore, research into novel, cost-friendly biomarkers that could improve the early detection of CKD and its progression in HIV aging populations is needed to allow early intervention.

## Figures and Tables

**Table 1:** Characteristics of study participants

Characteristics	Overall N = 278	PLWH, N = 139	No HIV, N = 139	P-value
	n (%)	n (%)	n (%)	
**Median Age in Years (IQR)**	66 (63–70)	66 (64–70)	65 (62–70)	0.06
**Sex**				0.15
Female	144 (51.8)	66 (47.5)	78 (56.1)	
Male	134 (48.2)	73 (52.5)	61 (43.9)	
**Highest level of education**				0.81
None-primary level	116 (41.7)	57 (41.0)	59 (42.5)	
secondary-tertiary level	162 (58.3)	82 (59.0)	80 (57.5)	
**Monthly household income in USDs**				0.61
≥ 1 USD per day	190 (68.4)	93 (66.9)	97 (69.8)	
≤ 1 USD per day	88 (31.6)	46 (33.1)	42 (30.2)	
**Smoking**				**0.01**
No	228 (82.0)	106 (76.3)	122 (87.8)	
Yes	50 (18.0)	33 (23.7)	17 (12.2)	
**Alcohol consumption**				0.15
No	197 (70.9)	104 (74.8)	93 (66.9)	
Yes	81 (29.1)	35 (25.2)	46 (33.1)	
**Non-HIV related comorbidities**				0.13
No chronic illness	54 (19.4)	22 (15.8)	32 (23.0)	
At least one chronic illness	224 (80.6)	117 (84.2)	107 (77.0)	
**Prior diagnosis of Hypertension**				0.54
No	117 (42.1)	56 (40.3)	61 (43.9)	
Yes	161 (57.9)	83 (59.7)	78 (56.1)	
**Prior diagnosis of Diabetes mellitus**				0.73
No	240 (86.3)	119 (85.6)	121 (87.0)	
Yes	38 (13.7)	20 (14.4)	18 (13.0)	
**Non-HIV related medications**				**0.03**
No chronic medication	75 (27.0)	30 (21.6)	45 (32.4)	
Anti-hypertensives and anti-diabetes medications	144 (51.8)	83 (59.7)	61 (43.9)	
Others	59(21.2)	26 (18.7)	33 (23.7)	
**Prior diagnosis of tuberculosis**				**<0.01**
No	219 (78.8)	80 (57.5)	139 (100.0)	
Yes	59 (21.2)	59 (42.5)	0 (0)	
**Median Systolic Blood Pressure (IQR)**	130 (121–140)	128 (118–134)	134 (125–150)	**<0.01**
**Median Diastolic Blood Pressure (IQR)**	82 (75–90)	80 (73–85)	86 (77–95)	**<0.01**
**Median Fasting Blood Sugar (IQR)**	5.6 (4.9–6.3)	5.6 (5.0–6.4)	5.4 (4.9–6.0)	**0.17**
**Body mass index**				**<0.01**
<18.5kg/m^2^	17 (6.1)	9 (6.5)	8 (5.8)	
18.5–25.0	134 (48.2)	81 (58.3)	53 (38.1)	
>25.0	127 (45.7)	49(35.2)	78 (56.1)	
**Proteinuria**				**<0.01**
Negative	190 (68.4)	78 (56.1)	112 (80.6)	
At least Trace proteinuria	88 (31.6)	61 (43.9)	27 (19.4)	

IQR = interquartile range, PLWH = people living with HIV, USD = US dollars, others = other medication apart from those used to treat diabetes mellitus and hypertension.

**Table 2:** Characteristics of PLWH in the study

HIV-related characteristics of older PLWH(N = 139)	n, (%)
**WHO clinical stage at ART**	
Stage I-II	27 (19.4)
Stage III-IV	112 (80.6)
**Median CD4 count (IQR)**	634 (486–917)
**Viral load**	
Detectable (> 50 copies/ml)	8 (5.8)
Undetectable (< 50 copies per ml)	131 (94.2)
**Current ART regimen**	
Others	3 (2.2)
Abacavir-based	44 (31.7)
TDF-based	92 (66.1)
**Ever used TDF**	
No	6 (4.30
Yes	133 (95.7)
**Median number of years on TDF (IQR)**	5 (4–9)
**Median number of years on ART (IQR)**	16 (11–18)

Supplementary table 1: PLWH = People living with HIV, TDF Tenofovir Disoproxil Fumarate, IQR = interquartile range

**Table 3:** Factors associated with kidney function impairment among people ≥ 60 years living with and without HIV

Characteristic of participants	Kidney function impairment, n (%)		Bivariate Analysis		Multivariate analysis	
	Yes: 64 (23%)	No: 214 (77%),	Unadjusted OR (Cl)	P-value	Adjusted OR (Cl)	P-value
**Median Age in Years (IQR)**	68 (65–74)	65 (63–69)	1.08 (1.00–1.17)	0.04	**1.13 (1.07–1.20)**	**<0.01**
**Sex**						
Male	28 (43.7)	106 (49.5)	1		1	
Female	36 (56.3)	108 (50.5)	1.72 (0.84–3.52)	0.14	**1.95 (1.06–3.62)**	**0.03**
**Alcohol consumption**						
No	50 (78.1)	147 (68.7)			1	
Yes	14 (21.9)	67 (31.3)	0.42 (0.17–1.05)	0.06	0.63 (0.29–14)	0.26
**HIV status**						
People without HIV	18 (28.1)	121 (56.5)	1		1	
Positive	46 (71.9)	93 (43.5)	3.32 (1.81–6.12)	<0.01	**3.89 (2.04–7.41)**	**<0.01**
**Prior diagnosis of hypertension** **						
No	20 (31.3)	97 (45.3)	1		1	
Yes	44 (68.7)	117 (56.7)	1.82 (1.00–3.30)	0.05	1.73 (0.91–3.93)	0.09
**Prior diagnosis of TB**						
No	43 (67.3)	176 (82.2)	1		1	
Yes	21 (32.8)	38 (17.8)	2.26 (1.21–4.24)	0.011	1.31 (0.62–2.80)	0.48

* Variables evaluated in a stratified analysis by HIV status only

** Variable was significant at multivariate analysis in PLWH in a stratified analysis by HIV status with OR = 2.19 (95%CI:1.02–4.67) and p-value = 0.04

## Data Availability

The de-identified data set will be available upon acceptance for publication of the manuscript with a request to the study team through the corresponding author.

## Abbreviations

AIDS: Acquired Immunodeficiency Syndrome
ART: Antiretroviral therapy
LICs: Low-income countries
CKD: Chronic Kidney Disease
CKD-EPI: Chronic Kidney Disease Epidemiology Collaboration
ESRD/ESKD: End-Stage Renal Disease/End-Stage kidney disease/kidney failure
eGFR: Estimated Glomerular Filtration Rate
HIVAN: HIV Associated Nephropathy
HIV: Human Immunodeficiency Virus
IDI: Infectious Diseases Institute
PLWH: People Living With HIV
TDF: Tenofovir disoproxil fumarate
